# The impact of surgical volume on hospital ranking using the standardized infection ratio

**DOI:** 10.1038/s41598-023-33937-y

**Published:** 2023-05-10

**Authors:** Shangyuan Ye, Daniel Li, Tingting Yu, Daniel A. Caroff, Jeffrey Guy, Russell E. Poland, Kenneth E. Sands, Edward J. Septimus, Susan S. Huang, Richard Platt, Rui Wang

**Affiliations:** 1 Department of Population Medicine, Harvard Pilgrim Health Care Institute and Harvard Medical School, Boston, MA 02215 USA; 2 Biostatistics Shared Resource, Knight Cancer Institute, Oregon Health and Science University, Portland, OR 97201 USA; 3 Department of Biostatistics, Harvard T. H. Chan School of Public Health, Boston, MA 02215 USA; 4 Department of Infectious Diseases, Lahey Hospital and Medical Center, Burlington, MA 01805 USA; 5 Clinical Operations Group, HCA Healthcare, Nashville, TN 37203 USA; 6 Texas A &M College of Medicine, Houston, TX 77030 USA; 7 University of California Irvine School of Medicine, Irvine, CA 92617 USA

**Keywords:** Scientific data, Statistics

## Abstract

The Centers for Medicare and Medicaid Services require hospitals to report on quality metrics which are used to financially penalize those that perform in the lowest quartile. Surgical site infections (SSIs) are a critical component of the quality metrics that target healthcare-associated infections. However, the accuracy of such hospital profiling is highly affected by small surgical volumes which lead to a large amount of uncertainty in estimating standardized hospital-specific infection rates. Currently, hospitals with less than one expected SSI are excluded from rankings, but the effectiveness of this exclusion criterion is unknown. Tools that can quantify the classification accuracy and can determine the minimal surgical volume required for a desired level of accuracy are lacking. We investigate the effect of surgical volume on the accuracy of identifying poorly performing hospitals based on the standardized infection ratio and develop simulation-based algorithms for quantifying the classification accuracy. We apply our proposed method to data from HCA Healthcare (2014–2016) on SSIs in colon surgery patients. We estimate that for a procedure like colon surgery with an overall SSI rate of 3%, to rank hospitals in the HCA colon SSI dataset, hospitals that perform less than 200 procedures have a greater than 10% chance of being incorrectly assigned to the worst performing quartile. Minimum surgical volumes and predicted events criteria are required to make evaluating hospitals reliable, and these criteria vary by overall prevalence and between-hospital variability.

## Introduction

The Centers for Medicare and Medicaid Services (CMS) Hospital-Acquired Condition Reduction Program (HACRP) is a pay-for-performance program that links Medicare payments to inpatient healthcare quality. This program requires the Secretary of Health and Human Services to impose a 1% payment reduction to the hospitals ranked in the worst performing quartile with respect to six quality measures^1^. One quality measure is the CMS recalibrated patient safety indicator 90, and the other five are Centers for Disease Control and Prevention (CDC) National Healthcare Safety Network healthcare-associated infection (HAI) measures^2^. These HAI measures are for central-line associated bloodstream infection, catheter-associated urinary tract infection, colon and hysterectomy surgical site infection (SSI), methicillin-resistant *Staphylococcus aureus* bacteremia, and clostridioides difficile infection.

All five of the HAI measures in the HACRP are evaluated using the CDC standardized infection ratio (SIR). The SIR is the primary summary measure used by the CDC National Healthcare Safety Network to track HAIs at a national, state, and local level over time, and is calculated by dividing the observed number of events by the predicted number of events^3^. The SIR adjusts for facility and patient-level factors, and is similar to the standardized mortality ratio (SMR), which is widely used by CMS and others in public health to analyze mortality data^4,5^.

Accurate hospital ranking is crucial for the success of HACRP. However, small surgical volumes and low event rates present methodological and statistical challenges that can impact the accuracy of these rankings^6^. Previous research has focused on the inverse association between surgical volume and surgical or mortality outcomes. Ross et al.^7^ found that acute myocardial infarction, pneumonia, and heart failure mortality rates were higher in lower-volume hospitals. Similar findings were reported in patients with sepsis^8^, acute pancreatitis^9^, and various gastrointestinal, cardiac, and vascular surgical procedures^10–12^. Concerns have been raised that accurate hospital ranking with the SIR may not be possible if surgical volumes are too small^13–15^. It also has been noted that the CMS SMR is more likely to flag hospitals with larger volumes as performing “worse than the US national rate”^16^. Caroff et al.^13^ found that the agreement between predicted SSI rates based on risk-adjustment models and observed SSI rates was moderate, with low procedure volumes and the small number of predicted events in individual hospitals being major limiting factors.

The accuracy of hospital rankings is affected not only by surgical volume, but also by the magnitude of infection rates and the level of heterogeneity in hospital-specific infection rates. The larger the heterogeneity in these rates, the easier it is to differentiate them. Small surgical volume and rare outcomes lead to a large amount of uncertainty in estimating hospital-specific SSI rates, making it more difficult to distinguish hospitals based on observed rates. Austin et al.^17^ defined a metric termed as ‘rankability’ which can be interpreted as the proportion of the variation between hospitals that is due to true differences in infection rates as opposed to sampling variation in the observed data. This rankability index ranges between 0 and 1, with higher values corresponding to better accuracy. When most of surgical volumes are small or the level of heterogeneity in true hospital-specific infection rates is small, the rankability will be low. While the rankability index provides an attractive overall measure of ranking accuracy for a given set of hospitals, it does not quantify ranking accuracy for each individual hospital relative to other hospitals in the pool of hospitals being ranked or provide a way to evaluate the minimal event requirements for reliable classification. To the best of our knowledge, such a tool is not currently available. This article aims to fill this gap and addresses the need for individualized accuracy metrics for each hospital and a means of evaluating the minimal event requirements for reliable classification.

In this article, we first define accuracy evaluation metrics such as power, false positive rate (FPR), positive predictive value (PPV), and negative predictive value (NPV) of identifying hospitals in the worst-performing quartile. We then propose a simulation-based algorithm to assess these metrics in real-world settings and to provide recommendations for the minimum surgical volumes required for reliable classification of hospitals into the worst-performing quartile, a crucial issue for Medicare penalties imposed by the HCARP. Through simulation studies, we evaluate the impact of surgical volume, the overall prevalence of the infection, variability in hospital-specific prevalence, as well as case-mix adjustment factors on these accuracy metrics.

The remainder of this article is organized as follows. The section “Models and classification accuracy measurements” introduces notation, models, and proposes accuracy evaluation metrics, as well as a simulation-based approximation algorithm for assessing these metrics in a given setting. In the section “Colon surgery surgical site infections”, we apply the proposed approach to a colon surgery SSI dataset to determine the number of predicted events and the surgical volume needed to reach a desired level of classification accuracy. The section “Simulation studies” reports simulation studies evaluating the performance of the proposed algorithm and assessing the impact of various factors on ranking accuracy metrics. We conclude with a discussion.

## Models and classification accuracy measurements

### Standardized infection ratio

Let $Y_{ij}$ denote the binary response variable of the *j*th patient in the *i*th hospital, and $\varvec{x}_{ij}$ denote the corresponding *p* dimensional vector of covariates with $i = 1, \ldots , m$, $j = 1, \ldots , n_i$, and $N=\sum _{i=1}^m n_i$. We assume that the outcome $Y_{ij}$ follows a Bernoulli distribution and consider the following generalized linear mixed effects model

1 $$\begin{aligned} \text{ logit }(p_{ij}) = \frac{\log (p_{ij})}{1 - \log (p_{ij})} = \alpha _i + \varvec{x}_{ij}^\top \varvec{\beta }, ~\alpha _i \sim N(\alpha , \sigma _\alpha ^2), ~p_{ij}=\text{ E }(Y_{ij} | \alpha _i, \varvec{x}_{ij}), \end{aligned}$$

where $\alpha _i$ is the intercept of hospital *i*, $\varvec{x}_{ij} = (x_{ij1}, \ldots , x_{ijp})^\top$ is a vector of patient specific covariates, and $\varvec{\beta }= (\beta _1, \ldots , \beta _p)^\top$ are the corresponding covariate effects. We further assume that the hospital-specific intercept $\alpha _i$s are independent and identically distributed with mean $\alpha$ and variance $\sigma _\alpha ^2$.

A hospital’s true ranking is determined by the value of $\alpha _i$, with larger values indicating worse performance. One way to rank hospitals is to use their standardized infection ratios (SIRs), defined as

2 $$\begin{aligned} \text{ SIR}_i = \frac{\sum _{j=1}^{n_i} Y_{ij}}{\sum _{j=1}^{n_i} \text{ expit } (\hat{\alpha }_s + \varvec{x}_{ij}^\top \hat{\varvec{\beta }}_s)} = \frac{Y_i}{\hat{\pi }_i}, \end{aligned}$$

where $\text{ expit }(a) = \frac{\exp (a)}{1 + \exp (a)}$ for $a \in \mathbb {R}$, $Y_i = \sum _{j=1}^{n_i} Y_{ij}$, $\hat{\pi }_i = \sum _{j=1}^{n_i} \text{ expit } (\hat{\alpha }_s + \varvec{x}_{ij}^\top \hat{\varvec{\beta }}_s)$, and $\hat{\alpha }_s$ and $\hat{\varvec{\beta }}_s$ are consistent estimates of $\alpha _s$ and $\varvec{\beta }_s$ in the model

3 $$\begin{aligned} \text{ logit }(p^*_{ij}) = \alpha _s + \varvec{x}_{ij}^\top \varvec{\beta }_s, ~p^*_{ij} = \text{ E }(Y_{ij} | \varvec{x}_{ij}). \end{aligned}$$

    Models in the form of (3) are usually referred to as marginal models or population-average models^18^. The parameters $\alpha _s$ and $\varvec{\beta }_s$ represent the population-averaged intercept and covariate effects, respectively. It has been shown that the parameters $(\alpha , \varvec{\beta }^\top )^\top$ in the model (1) are always larger (in absolute value) than the corresponding parameters $(\alpha _s, \varvec{\beta }_s^\top )^\top$ from the model (3), and that the relationship between $(\alpha _s, \varvec{\beta }_s^\top )^\top$ and $(\alpha , \varvec{\beta }^\top )^\top$ can be approximated using the cumulative Gaussian approximation to the logistic function^18,19^:

$$\begin{aligned} \alpha _s \approx \frac{\alpha }{\sqrt{c^2 \sigma _\alpha ^2 + 1}}, ~ \beta _{s,\ell } \approx \frac{\beta _\ell }{\sqrt{c^2 \sigma _\alpha ^2 + 1}} ~\text {for } \ell = 1, \ldots , p, \end{aligned}$$

where $$c = \frac{16 \sqrt{3}}{15 \pi }$$.

From the assumed logistic mixed effects model (1), conditioning on $\alpha _i$ and $\varvec{X}_i = (\varvec{x}_{i1}, \ldots , \varvec{x}_{in_i})$, we have $Y_i \sim \text {Poisson Binomial} (p_{i1}, \ldots , p_{in_i})$. The numerator $Y_i$ is the observed number of infections at hospital *i*, and the denominator $\pi _i$ represents the model-predicted number of infections for the same patients but treated at a “typical” hospital (i.e., with the infection probability representing the population average). Thus, hospitals with SIR greater than one are considered as “worse than average” and hospitals with SIR less than one are considered as “better than average”.

### Power, false positive rate, positive predictive number, and negative predictive number

To quantify the accuracy of classifying hospitals into the worst quartiles, we define several accuracy metrics. We define power as the probability of correctly being ranked in the worst quartile (SIR$_i$ in the upper quartile) given the hospital is truly in the worst quartile ($\alpha _i$ in the upper quartile), i.e.

$$\begin{aligned} \text{ Power}_i = P(\text{ SIR}_i \in \text {upper quartile } | \alpha _i \in \text {upper quartile}) = \frac{P(\text{ SIR}_i \in \text {upper quartile } \bigcap \alpha _i \in \text {upper quartile})}{P(\alpha _i \in \text {upper quartile})}. \end{aligned}$$

    We define FPR as the probability of erroneously being ranked in the worst quartile (SIR$_i$ in the upper quartile) given the hospital *i* is not in the worst quartile ($\alpha _i$ in the 1st–3rd quartile), i.e.

$$\begin{aligned} \text{ FPR}_i = P(\text{ SIR}_i \in \text {upper quartile } | \alpha _i \in \text {1st-3rd quartile}) = \frac{P(\text{ SIR}_i \in \text {upper quartile } \bigcap \alpha _i \in \text {1st-3rd quartile})}{P(\alpha _i \in \text {1st-3rd quartile})}. \end{aligned}$$

    We define PPV as the probability of truly being in the worst quartile ($\alpha _i$ in the upper quartile) given the hospital is being ranked in the worst quartile (SIR$_i$ in the upper quartile):

$$\begin{aligned} \text{ PPV}_i = P(\alpha _i \in \text {upper quartile } | \text{ SIR}_i \in \text {upper quartile}) = \frac{P(\text{ SIR}_i \in \text {upper quartile } \bigcap \alpha _i \in \text {upper quartile})}{P(\text{ SIR}_i \in \text {upper quartile})}. \end{aligned}$$

NPV is the probability of truly not being in the worst quartile ($\alpha _i$ in the 1st–3rd quartile) given the hospital is not being ranked in the worst quartile (SIR$_i$ in the 1st–3rd quartile):

$$\begin{aligned} \text{ NPV}_i = P(\alpha _i \in \text {1st-3rd quartile } | \text{ SIR}_i \in \text {1st-3rd quartile}) = \frac{P(\text{ SIR}_i \in \text {1st-3rd quartile } \bigcap \alpha _i \in \text {1st-3rd quartile})}{P(\text{ SIR}_i \in \text {1st-3rd quartile})}. \end{aligned}$$

    In practice, for a given dataset, since the true ranking of a hospital, the relative position of $\alpha _i$, is unknown, the power and FPR can be estimated for every hospital assuming that the hospital is in the worst quartile or not, respectively. The minimal predicted events (or surgical volume) threshold can be determined based on a pre-specified power or FPR threshold. On the other hand, because rankings based on SIR are available, we can estimate the PPV for hospitals being ranked in the worst quartile and the NPV for hospitals not being ranked in the worst quartile.

### Simulation-based approximation

For real-world settings based on an observed dataset, we can use a simulation-based algorithm to approximate the power, FPR, PPV, or NPV defined in the section “Power, false positive rate, positive predictive number, and negative predictive number”. Pseudocode for the proposed algorithm is provided in Algorithm 1. Because the true model parameters $(\varvec{\beta }^\top , \alpha , \sigma _\alpha ^2)^\top$ are unknown, we first fit a logistic mixed effects model to the data to obtain $(\hat{\varvec{\beta }}^\top , \hat{\alpha }, \hat{\sigma }_\alpha ^2)^\top$. We then simulate *K* datasets conditioning on the patient-level covariates $\varvec{X}$ and estimated parameter values $(\hat{\varvec{\beta }}^\top , \hat{\alpha }, \hat{\sigma }_\alpha ^2)^\top$, where $\varvec{X}= (\varvec{x}_{11}, \ldots , \varvec{x}_{m n_m})^\top$. That is, for the *k*th simulated dataset ($k=1,\dots , K$), we generate hospital effects and outcomes, denote by $\varvec{\alpha }^{(k)} = (\alpha _1^{(k)}, \ldots , \alpha _m^{(k)})^\top$ and $\varvec{Y}^{(k)} = (Y_{11}^{(k)}, \ldots , Y_{m n_m}^{(k)})^\top$, respectively, from model (1). The calculation of the SIR requires estimates of $(\varvec{\beta }_s^\top , \alpha _s)^\top$. If the published values (e.g., by CMS^3^) for these estimates are available, they can be used directly; otherwise, we can fit a logistic model (3) to obtain $(\hat{\varvec{\beta }}_s^\top , \hat{\alpha }_s)^\top$, and calculate **SIR**$^{(k)}$ =(SIR$_1^{(k)}, \ldots ,$ SIR$_m^{(k)}$)$^\top$ using Eq. (2).

Based on the terminology of measures of diagnostic accuracy^20^, we define the number of true positive (TP), true negative (TN), false positive (FP), and false negative (FN) for hospital *i* across the *K* simulated datasets as

$$\begin{aligned} N_{\text {TP}_i}= & {} \sum _{k=1}^K \left[ I(\alpha _i^{(k)}> \alpha _{0.75}^{(k)} \bigcap \text {SIR}_i^{(k)}> \text {SIR}_{0.75}^{(k)}) \right] , \\ N_{\text {TN}_i}= & {} \sum _{k=1}^K \left[ I(\alpha _i^{(k)} \le \alpha _{0.75}^{(k)} \bigcap \text {SIR}_i^{(k)} \le \text {SIR}_{0.75}^{(k)}) \right] , \\ N_{\text {FP}_i}= & {} \sum _{k=1}^K \left[ I(\alpha _i^{(k)} \le \alpha _{0.75}^{(k)} \bigcap \text {SIR}_i^{(k)}> \text {SIR}_{0.75}^{(k)}) \right] , \\ N_{\text {FN}_i}= & {} \sum _{k=1}^K \left[ I(\alpha _i^{(k)} > \alpha _{0.75}^{(k)} \bigcap \text {SIR}_i^{(k)} \le \text {SIR}_{0.75}^{(k)}) \right] , \end{aligned}$$

where $\alpha _{0.75}^{(k)}$ and SIR$_{0.75}^{(k)}$ are the 75th percentile of $\varvec{\alpha }^{(k)}$ and **SIR**$^{(k)}$, respectively. Power, FPR, PPV, and NPV can be estimated by

4 $$\begin{aligned} \widehat{\text {Power}}_i = \frac{N_{\text {TP}_i}}{N_{\text {TP}_i} + N_{\text {FN}_i}}, ~\widehat{\text {FPR}}_i = \frac{N_{\text {FP}_i}}{N_{\text {FP}_i} + N_{\text {TN}_i}}, ~\widehat{\text {PPV}}_i = \frac{N_{\text {TP}_i}}{N_{\text {TP}_i} + N_{\text {FP}_i}}, ~\widehat{\text {NPV}}_i = \frac{N_{\text {TN}_i}}{N_{\text {TN}_i} + N_{\text {FN}_i}}, \end{aligned}$$

for $$i = 1, \ldots , m$$.
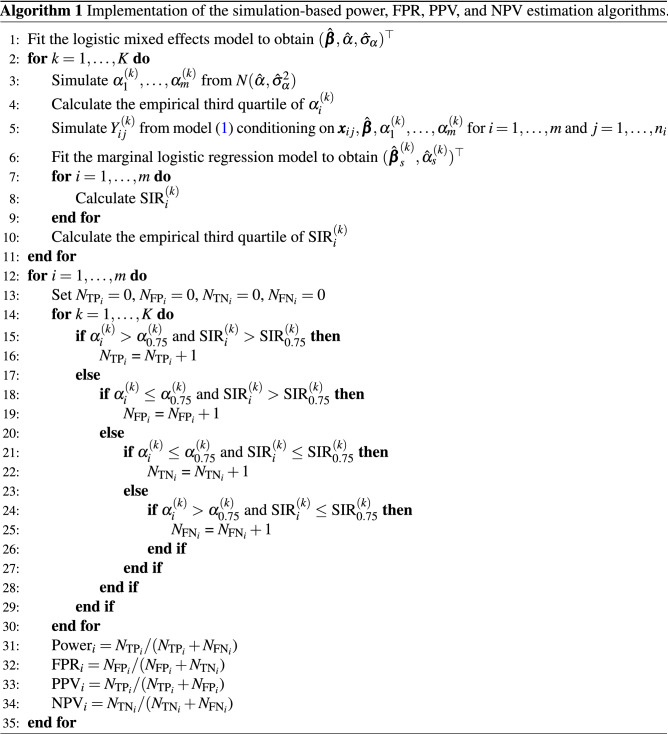


## Colon surgery surgical site infections

Colon surgery is one of the most commonly performed procedures in U.S. hospitals. Colorectal SSI is one of the HAI measures used in the HACRP to determine hospital reimbursement. But the impact of surgical volume on the accuracy of classifying hospitals into the worst quartile has not been well quantified. Currently, hospitals with less than one expected SSI are excluded from rankings^3^, but whether or to what extent this exclusion criterion is an effective approach is unknown.

We apply the proposed algorithm (Algorithm 1) to calculate the power, FPR, PPV, and NPV associated with being ranked in the worst quartile for hospitals in the HCA colon surgery SSI dataset described in Caroff et al.^13^ The dataset included 39,468 adult patients who underwent colon surgery within 149 facilities affiliated with HCA Healthcare from January 2014 through December 2016. Only the first eligible episode of colon surgery for each individual was included. The number of surgical volumes in each hospital ranged from 2 to 903. Colon surgery SSIs were determined by each hospital’s infection prevention staff using CDC National Healthcare Safety Network criteria^21^. A total of 1216 (3.1%) of patients developed deep incisional or organ/space SSI. Patient and hospital level data were obtained from the CDC National Healthcare Safety Network submissions and the HCA central data repository.

**Figure 1:**
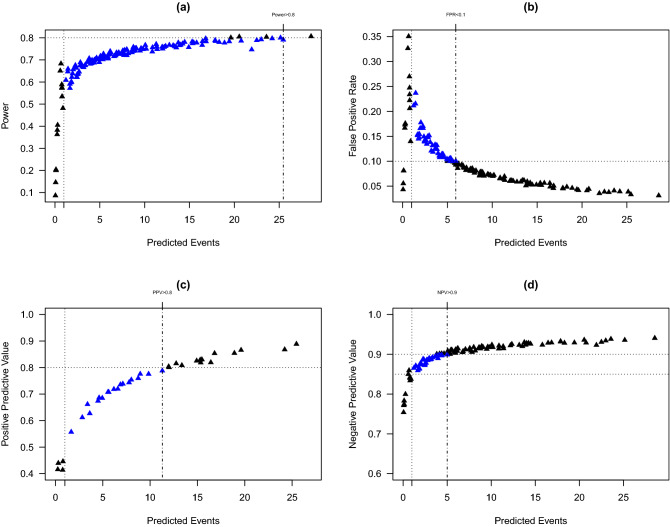
Estimated classification accuracy measures by the number of predicted events. Each hospital corresponds to a triangle. (**a**) Estimated power for all hospitals. Hospitals highlighted in blue correspond to those with the number of predicted events greater than 1 and power less than 80%. (**b**) Estimated false positive rate (FPR) for all hospitals. Hospitals in blue correspond to those with the number of predicted events greater than 1 and FPR higher than 10%. (**c**) Estimated positive predictive value (PPV) for hospitals being ranked into the worst quartile. Hospitals in blue correspond to those with the number of predicted events greater than 1 and PPV lower than 80%. (**d**) Estimated negative predictive value (NPV) for hospitals not being ranked into the worst quartile. Hospitals in blue correspond to those with the number of predicted events greater than 1 and NPV lower than 90%.

We consider rankings based on the current CMS model, where age, gender, ASA (American Society of Anesthesiologists) score, diabetes, BMI (Body Mass Index), and primary closure are included as covariates. Figure 1a,b present the number of predicted events against approximated power and FPR for all hospitals ($n = 149$). Results are based on 10,000 simulated datasets ($K = 10,000$). As the number of predicted events increases, power generally increases while FPR generally decreases. Based on the CDC exclusion criteria, 15 hospitals with predicted events < 1 would be excluded from ranking. However, among 134 hospitals with predicted events $\ge 1$, only four hospitals are associated with at least 80% chance of being correctly classified into the worst quartile if they are truly in that quartile. The minimum number of predicted events to achieve $\ge$ 80% power is 25.5. Fifty hospitals with predicted events $\ge$ 1 are associated with an FPR greater than 10%. The minimum number of predicted events to achieve $\le$ 10% FPR is 6.0 events.

Figure 1c presents the estimated PPV for the hospitals ($n = 37$) being ranked in the worst quartile. Nineteen hospitals with predicted events $\ge 1$ have PPV less than 80% (blue triangles). The minimal number of predicted events to achieve $\ge$80% PPV is 11.3 events. Figure 1d presents the estimated NPV for the hospitals ($n = 112$) not being ranked in the worst quartile. All hospitals with predicted events $\ge 1$ have PPV greater than 85%, and among these hospitals, 31 have PPV less than 90% (blue triangles). The minimal number of predicted events to achieve $\ge$ 90% NPV is 5.0 events.

**Figure 2:**
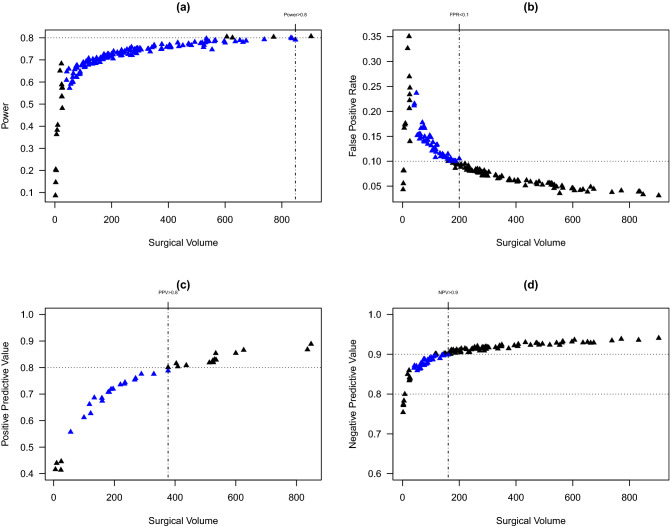
Estimated classification accuracy measures by the hospital surgical volume ($n_i$). Each hospital corresponds to a triangle. (**a**) Estimated power for all hospitals. Hospitals highlighted in blue correspond to those with the number of predicted events greater than 1 and power less than 80%. (**b**) Estimated false positive rate (FPR) for all hospitals. Hospitals in blue correspond to those with the number of predicted events greater than 1 and FPR higher than 10%. (**c**) Estimated positive predictive value (PPV) for hospitals being ranked into the worst quartile. Hospitals in blue correspond to those with the number of predicted events greater than 1 and PPV lower than 80%. (**d**) Estimated negative predictive value (NPV) for hospitals not being ranked into the worst quartile. Hospitals in blue correspond to those with the number of predicted events greater than 1 and NPV lower than 90%.

Figure 2 presents the estimated classification accuracy measures by the hospital surgical volume. To achieve a power of greater than 80%, a FPR of less than 10%, an 80% PPV, or a 90% NPV, the surgical volume needs to exceed 848, 200, 377, or 161, respectively.

## Simulation studies

We perform simulation studies to assess the performance of the proposed simulation-based algorithm and to investigate the impact of the overall event rate, between-hospital heterogeneity, and model misspecification on the four ranking accuracy metrics defined in the section “Power, false positive rate, positive predictive number, and negative predictive number”.

### Data generation processes

We generate data mimicking the structure of the HCA colon surgery SSI data, where the intraclass correlation coefficients (ICC) for each covariate range between 0.0066 and 0.1211, reflecting a modest level of heterogeneity in patient population across hospitals. The Pearson’s correlation coefficients among these covariates range from $-0.2722$ to 0.6515.

Outcomes are generated based on the generalized mixed effects model (1). For most simulation studies except in the section “Effect of underfitting”, we consider the CMS model with the six risk factors used in the section “Colon surgery surgical site infections” as the true outcome data-generating model. When evaluating the impact of underfitting, we use the Claims-plus-EHR model derived in Caroff et al.^13^ which included additional risk factors as the true outcome data generating model. We fit a generalized mixed effects model on the HCA colon surgery SSI data and use the fitted coefficients as the true parameter values in the data-generating process. The covariate ICCs and corresponding coefficients are summarized in Table 1. The random effects $(\alpha _1, \ldots , \alpha _m)$ are generated from a Normal distribution with mean $\alpha = -2.7862$ and variance $\sigma _\alpha ^2 = 0.5^2$.

**Table 1:** Covariates and corresponding coefficients.

Name	Coefficient ($\beta$)	ICC
$X_1$: Age (year)	− 0.0087	0.0732
$X_2$: Gender (male)	0.0361	0.0229
$X_3$: ASA score (1, 2, 3, 4, 5)	0.1470	0.1123
$X_4$: Diabetes (yes)	0.1181	0.0230
$X_5$: BMI ($\ge 30$)	0.4235	0.0166
$X_6$: Closure technique (other)	− 0.9813	0.0433

### Performance of the proposed simulation-based algorithm

We first assess the performance of our proposed simulation-based algorithm. For each dataset, outcomes are generated conditioning on the observed covariates from the HCA colon surgery SSI data. We apply the Algorithm 1 with $K = 1000$ and compare the resulting power, FPR, PPV, and NPV estimates with the empirical true values. To obtain these empirical true values, we simulate 10,000 datasets based on the true parameter values and calculated the corresponding SIRs. For each hospital, the empirical power, FPR, PPV, and NPV are calculated as in (4).

**Figure 3:**
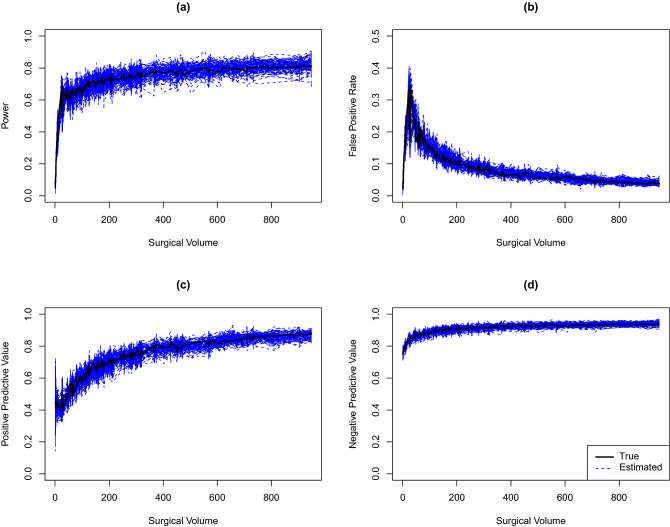
True and estimated power (**a**), false positive rate (**b**), positive predictive value (**c**), and negative predictive value (**d**) by surgical volume. True accuracy measures are obtained from simulation (black curves). The corresponding estimates for each of the 100 simulated datasets are obtained by the proposed algorithm (blue curves).

Figure 3 presents the true and estimated accuracy measures from 100 simulated datasets. Estimates from the algorithm (100 blue dashed curves) are close to and centered at the corresponding true values (solid black curve) for all measures, indicating our proposed algorithm can provide accurate estimates of the true parameter values.

### Impact of the overall event rate and the random effects variance

A key driver of the accuracy of hospital rankings is the level of heterogeneity in the true hospital-specific infection rates. The expectation of the empirical variance ($s^2$) of hospital-level event rate is^22^

5 $$\begin{aligned} \text{ E }(s^2) = \frac{\pi (1-\pi )}{\bar{n}_H} + \sigma _\alpha ^2, \end{aligned}$$

where $\pi$ is the overall event rate and $\bar{n}_H$ is the harmonic mean of surgical volumes. The expectation in Eq. (5) increases as $\sigma ^2_{\alpha }$ increases and is maximized when $\pi =0.5$ for a fixed $\sigma ^2_{\alpha }$. A related concept is “rankability” (or “reliability”), which is defined as

$$\begin{aligned} r = \frac{\sigma _\alpha ^2}{\sigma _\alpha ^2 + \text{ median }(s_i^2)}, \end{aligned}$$

where $s_i$ represents the sampling standard error of the observed hospital-specific infection rates for the *i*th hospital^17,23^. Both $\text{ E }(s^2)$ and *r* provide an overall measure of ranking accuracy for a given set of hospitals. The metrics we define and investigate in this article aim to provide a tool to quantify ranking accuracy for each individual hospital relative to other hospitals in the pool of hospitals being ranked and to enable us to assess the role of surgical volume (hospital-specific characteristics) in combination with other important contributing factors such as the overall event rate and between hospital heterogeneity on classification accuracy.

#### Impact of overall event rate

In the colon SSI setting described in the section “Colon surgery surgical site infections”, the overall event rate is about $3\%$. We evaluate the impact of the overall event rate on hospital ranking accuracy by increasing the random effects mean $\alpha$, representing the overall event rate, to $5\%$, $10\%$, $15\%$, $20\%$, $30\%$, and $50\%$. In order to preserve the heterogeneous patient populations across hospitals and the correlation structure among covariates, for each simulated dataset, we re-sample covariates with replacement from each hospital. Outcomes are generated as described in the section “Data generation processes”. The empirical power, FPR, PPV, and NPV are calculated based on 10,000 simulated datasets.

**Figure 4:**
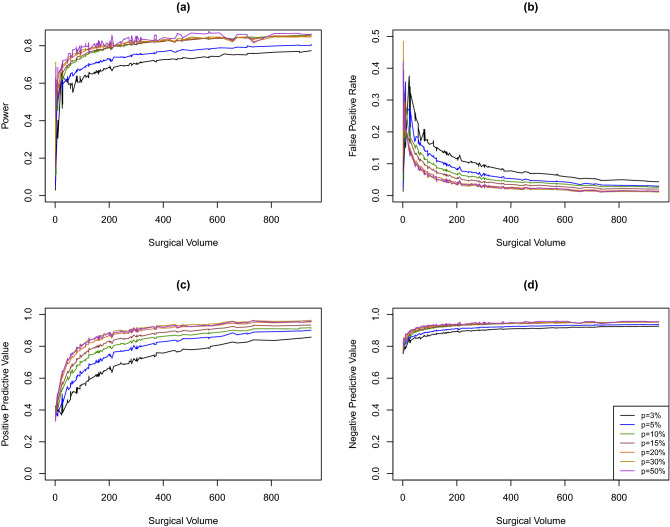
Empirical power (**a**), false positive rate (**b**), positive predictive value (**c**), and negative predictive value (**d**) by surgical volume for varying overall event rates.

**Figure 5:**
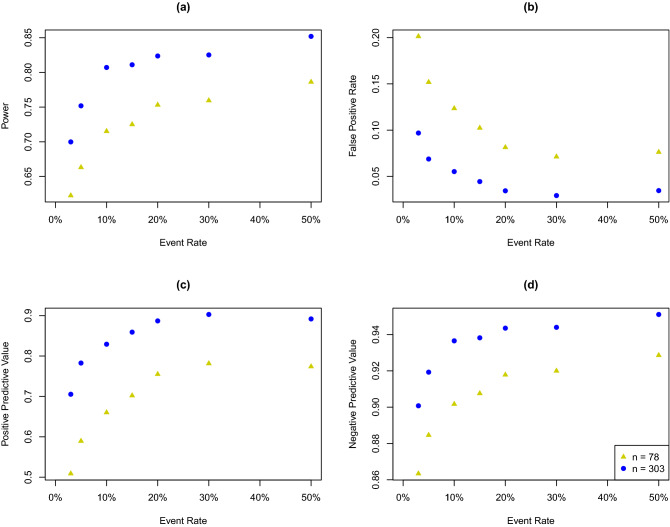
Empirical power (**a**), false positive rate (**b**), positive predictive value (**c**), and negative predictive value (**d**) by overall event rates for two individual hospitals.

Empirical power, FPR, PPV, and NPV by surgical volume for different overall event rates are presented in Fig. 4. Generally, a higher overall event rate (up to 50%) is associated with higher ranking accuracy: higher power, PPV, and NPV, as well as lower FPR. The magnitude of improvement becomes smaller when the overall event rate increases to 15%. As an illustration, we present the accuracy measures by the overall event rate for two hospitals with surgical volumes 78 (yellow triangles) and 303 (blue solid circles) in Fig. 5.

#### Impact of random effects variance

We assess the impact of between-hospital heterogeneity by increasing the random effects variance to $\sigma _\alpha ^2 = 0.75^2, 1.0^2$. Similar the simulation study in the section “Impact of the overall event rate and the random effects variance”, we calculate the empirical power, FPR, PPV, and NPV based on 10,000 simulated datasets.

**Figure 6:**
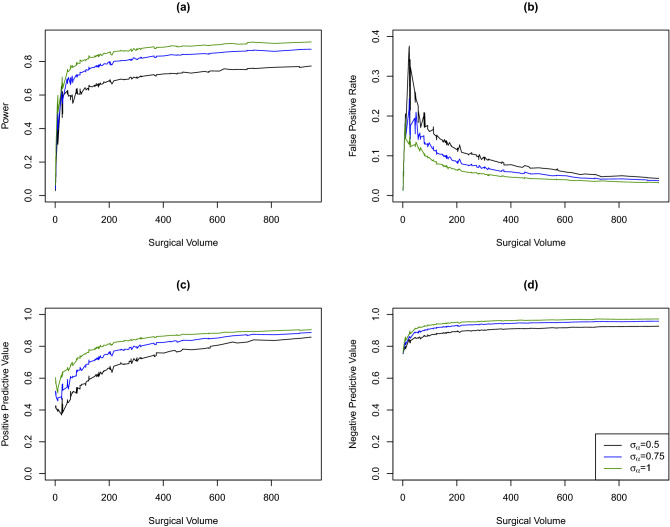
Empirical power (**a**), false positive rate (**b**), positive predictive value (**c**), and negative predictive value (**d**) by surgical volume for different random effects variances.

Results are presented in Fig. 6. As expected, a larger between-hospital heterogeneity is associated with increased power, PPV and NPV, and decreased FPR.

### Impact of model misspecification

Our next set of simulation studies investigates the impact of risk-adjustment model misspecification on ranking accuracy. We focus on two scenarios: (1) model overfitting, that is, the risk-adjustment model includes additional covariates that are not risk factors for the outcome; and (2) model underfitting, that is, the risk-adjustment model misses important risk factors for the outcome.

#### Effect of overfitting

**Figure 7:**
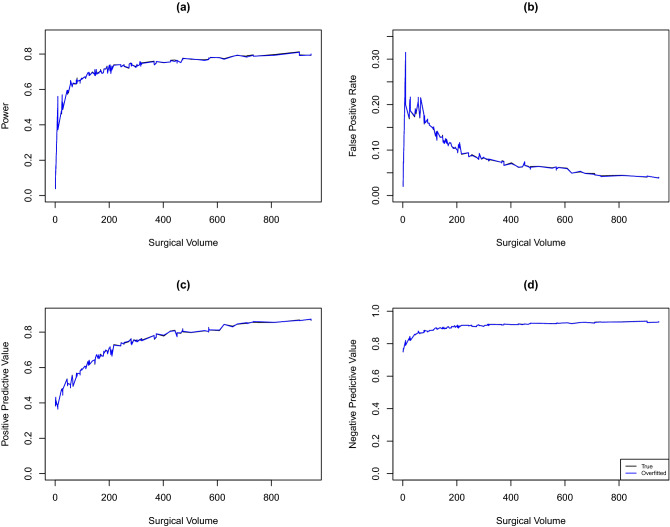
Empirical power (**a**), false positive rate (**b**), positive predictive value (**c**), and negative predictive value (**d**) by hospital volume when the CMS model was set as the correct model.

We first evaluate the effect of including additional covariates that are unrelated to the outcome into the risk-adjustment model after the set of risk factors have been included. The true outcome model is set as the CMS model with the coefficients $\varvec{\beta }, \alpha$, and $\sigma _\alpha ^2$ estimated from the observed data. We generate 10,000 datasets and calculate the SIRs based on CMS model (correct model) and Claims-plus-EHR model (overfitted model). Results of empirical power, FPR, PPV, and NPV are summarized in Fig. 7. The ranking accuracy curves based on the true and overfitted models overlap, suggesting that classifying hospitals into the worst quartile based on an overfitted model has negligible effect on the ranking performance.

#### Effect of underfitting

**Figure 8:**
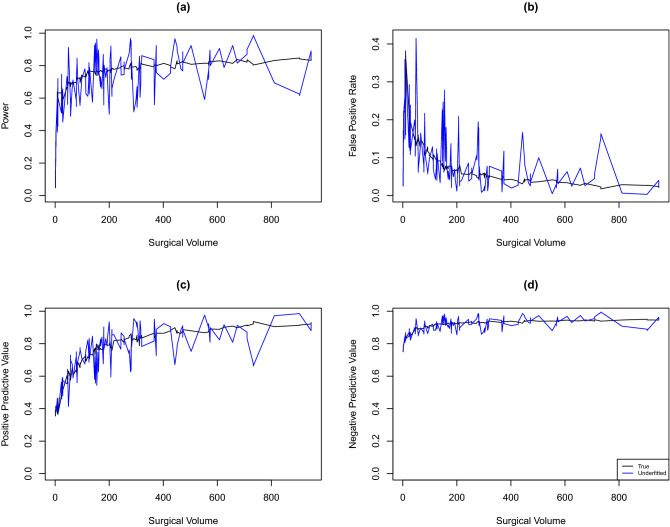
Empirical power (**a**), false positive rate (**b**), positive predictive value (**c**), and negative predictive value (**d**) by hospital volume when the Claims-plus-EHR model was set as the correct model.

To assess the effect of model underfitting, we set the Claims-Plus-EHR model developed in Caroff et al.^13^ as the true model. The Claims-Plus-EHR model includes laparoscopy, age, ASA score, diabetes status, BMI, sex, Charlson/Elixhauser comorbidities, concomitant colon procedures, concomitant noncolon intraabdominal procedures, anesthesia, procedure duration, wound class, and use of primary closure as covariates. We generate outcomes from the Claims-plus-EHR model, where the corresponding coefficients, $\alpha$ and $\sigma _\alpha ^2$ are estimated from the observed data. We generate 10,000 datasets and calculate SIRs based on the Claims-plus-EHR model (correct model) and the CMS model (underfitted model), respectively. Results are summarized in Fig. 8. We observe that the power, FPR, PPV and NPV curves based on the underfitted model (i.e., omitting important risk factors) can be substantially higher or lower compared to their empirical true values based on the correct model that fully adjusts the case-mix.

## Discussion

Motivated by the CMS HACRP, we investigate the effect of hospital volume on identifying hospitals in the worst-performing quartile. We define accuracy measures to quantify classification accuracy and propose simulation-based algorithms that approximate the power, FPR, PPV, and NPV associated with being classified into the worst-performing quartile.

Mimicking data from HCA healthcare, we perform simulation studies to investigate the impact of surgical volume, the overall event rate, between-hospital heterogeneity, and risk-adjustment on classification accuracy. Our results show hospital ranking accuracy is affected by several factors. Different outcomes have different overall event rates and different between-hospital variability in observed event rates. All these factors in addition to the distribution of volumes for the set of hospitals being evaluated affect ranking accuracy^24,25^. For any combination of outcome and quality measure, the proposed simulation-based algorithm can account for all these factors and help identify which hospitals can and cannot be accurately ranked.

We find that as hospital surgical volume increases, the power, PPV, and NPV generally increase and the FPR generally decreases. These general patterns are observed for overall event rates from 3 to 50%, and such event rates are representative of a wide variety of medical conditions. For example, 30-day mortality rates among 2004–2006 Medicare patients ranged from 10 to 20% for acute myocardial infarction, pneumonia, and heart failure^7^. Furthermore, 30-day mortality rates among 2000–2009 Medicare patients ranged from 6 to 14% for gastrointestinal procedures, 3.5–12.5% for cardiac procedures, and 3–6% for carotid endarterectomy^11^.

Our results suggest that current minimum hospital volume and predicted events criteria may be insufficient. When evaluating HAIs, the CDC only calculates SIRs for hospitals with predicted events $\ge 1$^3^. When evaluating 30-day mortality and readmission events, CMS only requires the hospital volume to be $\ge 25$ (https://www.medicare.gov/care-compare/). These criteria are applied to all medical events regardless of other factors. However, our results show that power, FPR, PPV, and NPV are also affected by overall event rates and between-hospital variability. For example, as illustrated in Fig. 5, for a hospital surgical volume of 78, the power for an event with an overall rate 3% would be $\approx$ 62%, but the power for an event with an overall rate 20% would be $\approx$ 75%. In addition, the SIR criteria of $\ge 1$ predicted events may be inadequate; applying our algorithm to the HCA colon SSI dataset, the minimum number of predicted events to achieve $\ge$ 80% power or $\le$ 10% FPR is 25.5 and 6.0 events, respectively. Our simulation results based on datasets mimicking HCA data indicate that missing important covariates in the risk-adjustment models can lead to inaccurate power, FPR, PPV, and NPV approximations. This underscores the importance of appropriate variable selection in constructing a proper risk-adjusted model.

There are some limitations with our study. While CMS HACRP flags hospitals with the lowest quartile HAI measures, different programs have different methods for identifying poorly performing hospitals. For example, CMS identifies hospitals with subpar 30-day mortality and readmission criteria by looking at 95% confidence intervals for the standardized mortality ratio. The model used in our simulation analyses is only an approximation of reality, and the patient covariates used in studying colon surgery SSI are likely different for other medical outcomes. However, regardless of the quality measure and outcome being studied, the proposed algorithm can be adapted to evaluate the ranking accuracy for a given set of hospitals and to identify minimum surgical volume criteria in other settings. The finding that overall event rates and between-hospital variability affect hospital ranking performance is also generalizable to other quality measures such as the standardized mortality ratio and to other medical and surgical outcomes.

In conclusion, we develop a simulation-based algorithm to estimate the classification accuracy of ranking hospitals into the worst-performing quartile based on the SIR. This algorithm can help us determine the minimum hospital surgical volume requirements and predicted event cutoffs for a particular setting. The results from applying the proposed algorithm to the HCA colon surgery SSI dataset suggest that, among 37 facilities being ranked in the worst quartile, those facilities that performed fewer than 377 procedures in the 3-year period had at least a 20% probability of being incorrectly ranked in the worst quartile. This highlights the importance of adequate surgical volume for accurate hospital profiling. Based on data from prior work^26^, 3934 US hospitals performed colon surgery on fee-for-service Medicare beneficiaries in the 3-year period of 2010–2012. When limited to Medicare beneficiaries only, 3236 (82%) performed less than 200 total colon procedures during this period. The minimum surgical volume criteria for ranking and profiling hospitals ideally should vary by overall event rates and between hospital variability, as ranking accuracy is significantly affected by both factors. When the minimum hospital surgical volume requirements are not met, one may consider delaying the timing of ranking until an adequate number of surgical procedures have been performed. Although we focus on healthcare-acquired infections and the SIR in our study, our conclusions and tools developed are broadly applicable to other quality measures and outcomes. Such modifications to minimum hospital volume criteria could prevent unmerited financial penalties for hospitals and improve the accuracy of existing CMS hospital evaluation programs.

## Data Availability

The R code to implement the proposed algorithm and an illustration based on a simulated dataset are provided at https://github.com/shyye008/Hospital-ranking. The colon surgical infection SSI data used in the section “Colon surgery surgical site infections” are not available due to privacy and ethical concerns.
